# Food-derived protein-based calcium chelates: digestive stability, intestinal transport, and bone-related functions

**DOI:** 10.3389/fnut.2026.1857619

**Published:** 2026-08-13

**Authors:** Tingting Li, Yaxiong Gao, Qiang Zhou, Yuan Xie, Xingwen Xie, Wenjie Wei

**Affiliations:** 1 Gansu Provincial Maternity and Child Health Hospital, Lanzhou, China; 2 Affiliated Hospital of Gansu University of Chinese Medicine, Lanzhou, China; 3 Gansu Provincial Hospital of TCM, Lanzhou, China; 4 Northwest Normal University, Lanzhou, China

**Keywords:** calcium bioaccessibility, gastrointestinal digestion, gut–bone axis, intestinal transport, osteoporosis, peptide–calcium chelates, protein-based calcium chelates

## Abstract

Conventional calcium supplements often show limited bioaccessibility and suboptimal gastrointestinal tolerance. Food-derived proteins, protein hydrolysates, and peptide–calcium chelates have been investigated as protein-based calcium chelates. This review organizes these systems along a structural continuum from proteins to hydrolysates and peptide–calcium chelates, in which enzymatic hydrolysis, fractionation, purification, and chelation reshape carrier composition, molecular weight distribution, and calcium coordination characteristics. Within this framework, this review examines the preparation strategies, molecular features, gastrointestinal stability, intestinal transport behavior, and bone-related functions of protein-based calcium chelates. Particular attention is given to the effects of chelate type, molecular weight distribution, peptide sequence, and coordination stability on calcium retention, solubility, and transport-related behavior during digestion, as well as their links with osteogenic responses, gut microbiota changes, and the gut–bone axis. Overall, this review provides a structured framework for evaluating food-derived protein-based calcium chelates and their potential application in food-based calcium delivery systems, while highlighting future needs in functional validation, product standardization, safety assessment, and clinically relevant evidence.

## Graphical abstract

**Figure:**
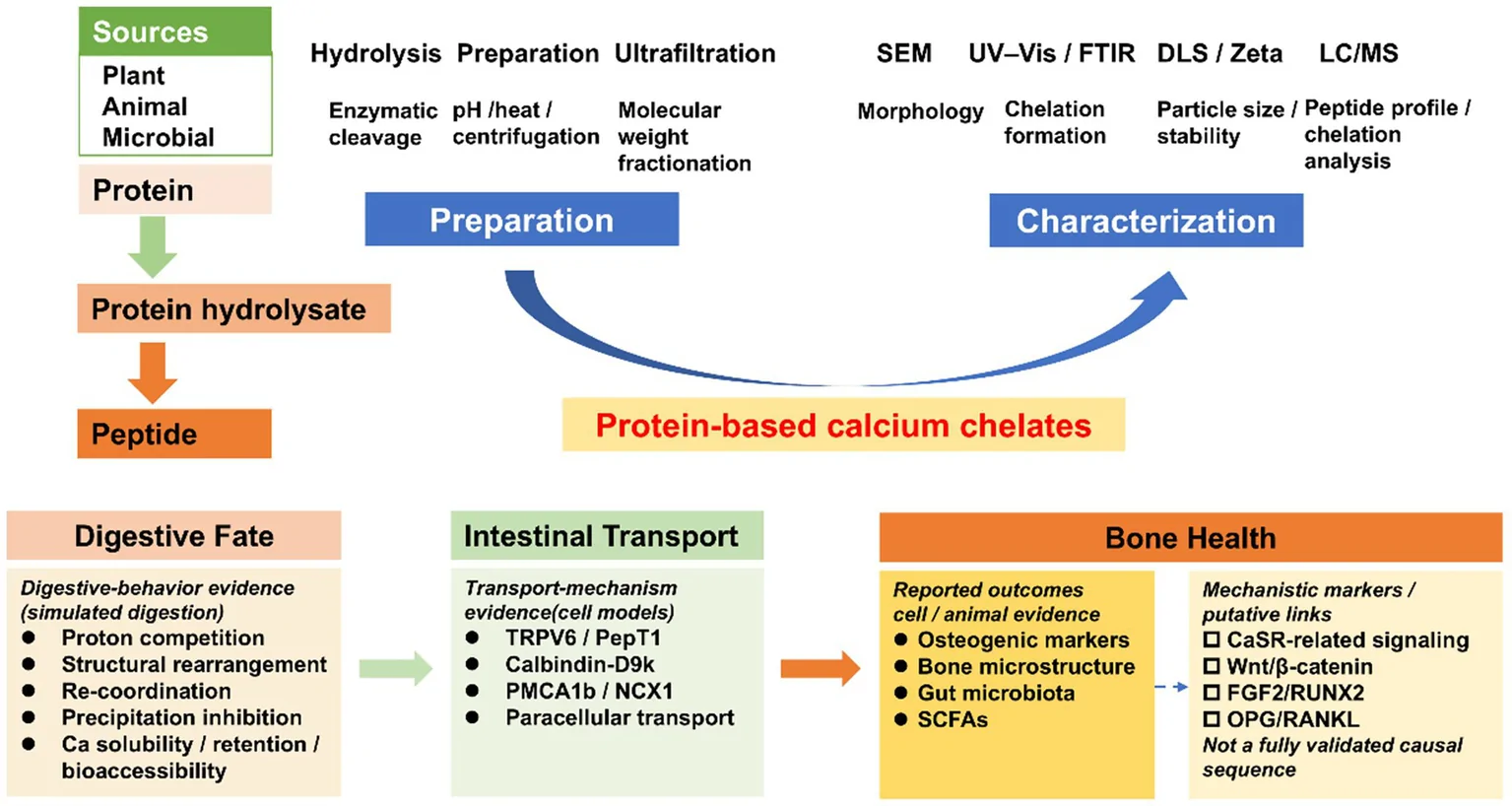

## 1 Introduction

Global population aging is accelerating, with the number of people aged 60 years and older projected to reach 2.1 billion by 2050, and approximately two-thirds of this population are expected to live in low- and middle-income countries. This demographic transition is expected to further increase the public health burden of age-related disorders, including osteoporosis, a major bone health concern characterized by low bone mass, microarchitectural deterioration, and increased fracture risk (1). Osteoporosis affects approximately 200 million people worldwide, and its prevalence increases markedly with age (2, 3). Although anti-osteoporotic drugs such as bisphosphonates remain important in clinical management, concerns regarding long-term safety and poor adherence have increased interest in complementary nutritional strategies (4). Calcium supplementation remains a cornerstone approach; however, conventional calcium salts still have practical limitations, including reduced utilization under low gastric acid conditions, gastrointestinal discomfort, and interference from dietary antinutritional factors such as oxalates and phytates (5–9). These limitations suggest that calcium supplementation should be considered not only in terms of intake level, but also in relation to calcium form, digestive stability, and subsequent intestinal uptake and utilization (10–12). In this context, food-derived proteins, protein hydrolysates, and peptide–calcium chelates have been explored as food-derived carriers for improving calcium delivery (13–16).

The calcium-binding behavior of food-derived proteins is closely related to amino acid composition, calcium-binding residues, phosphorylation status, protein conformation, and source-dependent structural features. Casein is a representative animal protein with strong calcium-carrying capacity because its phosphoserine clusters (Ser-P) provide densely negatively charged coordination regions (17). Collagen and bone-derived proteins also provide side-chain carboxyl groups that may participate in calcium interaction, although many studies focus on their hydrolysates or derived peptides rather than intact collagen itself (14, 18, 19). Some plant-derived proteins, such as soy and wheat, show different calcium-binding behavior from highly phosphorylated animal proteins, partly because of differences in phosphorylation, conformation, and matrix composition (20, 21). Beyond these intrinsic properties, enzymatic hydrolysis is a key step in converting protein matrices into more accessible calcium-binding systems (22, 23). Different proteases, such as Alcalase and Flavorzyme, generate distinct peptide profiles, while hydrolysis degree affects molecular weight distribution and coordination stability (24, 25). Controlled hydrolysis can enrich low-molecular-weight calcium-binding peptides, whereas over-degradation may disrupt cooperative binding motifs or generate fragments with insufficient coordination stability (26–28). Subsequent ultrafiltration or chromatographic separation can further enrich peptide fractions with stronger calcium-binding capacity (23, 29). Accordingly, the calcium-carrying behavior of protein hydrolysates reflects both the intrinsic sequence features of the parent proteins and the peptide-release patterns imposed by hydrolysis. Dairy-derived hydrolysates often retain phosphoserine-rich motifs and therefore usually display high calcium affinity (30, 31), whereas marine protein hydrolysates rely mainly on acidic residues, especially aspartic acid (Asp) and glutamic acid (Glu), for calcium coordination (32, 33). In plant-derived systems, enzymatic treatment combined with physical-field processing may improve calcium-binding performance by increasing the exposure of potential binding groups (27, 34, 35).

Beyond source-dependent differences and hydrolysis-mediated peptide release, increasing attention has been paid to the structural stability and biological functions of peptide–calcium chelates after chelate formation. Once formed, these chelates may develop coordination-stabilized structures and display several potential advantages (30, 36). First, they can improve gastrointestinal stability by maintaining calcium in a more soluble state under intestinal conditions and reducing the formation of insoluble precipitates (31, 37, 38). Second, compared with inorganic calcium salts, peptide–calcium chelates may participate in absorption through multiple transport-related processes, potentially involving pathways associated with peptide transporter 1 (PepT1), Calbindin-D9k, and other calcium transport-related proteins, although the relative contribution of each pathway appears to be system-dependent (14, 28, 39). Third, some systems may function not only as calcium chelates but also as sources of bioactive peptides with osteoregulatory potential (40, 41). For example, casein phosphopeptides have been reported to influence osteoblast proliferation, differentiation, calcium uptake, and mineralization in cell models (42). Fish-bone-derived peptide–calcium systems have also been associated with improved calcium absorption-related indicators and bone-related parameters in animal models (43, 44). Therefore, the value of peptide–calcium chelates lies not only in calcium delivery itself, but also in their potential dual role as mineral carriers and bioactive peptide systems.

Literature searches were performed in the Web of Science database, primarily covering publications from 2020 to 2026, with earlier foundational studies included where necessary to support mechanistic background. The core search terms included “food-derived protein–calcium chelate,” “protein hydrolysate–calcium chelate,” “peptide–calcium chelate,” “calcium supplementation,” “gastrointestinal digestion,” “intestinal transport,” “gut–bone axis,” and “bone health regulation.” Eligible studies included peer-reviewed original articles and relevant reviews on food-derived proteins, protein hydrolysates, or peptide–calcium chelates, particularly those addressing calcium binding, chelate preparation, structural characterization, gastrointestinal stability, calcium solubility or bioaccessibility, intestinal transport, bone-related outcomes, gut microbiota responses, or translational limitations. Studies were excluded or not emphasized if they were unrelated to food-derived protein-based calcium carriers, focused on non-food-derived synthetic chelators or unrelated mineral systems, lacked direct relevance to calcium binding, digestion, transport, or bone-related outcomes, or were non-peer-reviewed materials, conference abstracts, patents, theses, or non-research reports. The review mainly considered English-language publications; non-English studies were not used as primary evidence unless sufficient English-language information was available for reliable interpretation. This search strategy was intended to support a critical narrative synthesis rather than a formal systematic review or meta-analysis.

Several related reviews have summarized calcium supplements and peptide–calcium chelates (45), casein phosphopeptides (46), calcium–peptide chelates (30), or mineral ion-chelating peptides (47), with emphasis on preparation, structure–activity relationships, absorption pathways, and bioavailability-related evaluation. However, these reviews mainly focus on specific peptide chelates, casein-derived systems, or broader mineral-chelating peptides. In contrast, the present review emphasizes the structural progression from intact food proteins to hydrolysates and peptide–calcium chelates, and links this progression with digestive stability, intestinal transport, bone-related functions, and gut–bone axis-related responses.

Based on this scope, this review focuses on food-derived proteins, protein hydrolysates, and peptide–calcium chelates as a structural continuum of calcium-binding systems. This review examines amino acid sequence, calcium-binding motifs, higher-order conformation, and molecular weight distribution as key structural factors related to coordination behavior and chelation stability across different hierarchical levels. It also summarizes gastrointestinal behavior, especially calcium retention, solubility, and bioaccessibility, and reviews recent advances in intestinal absorption, osteogenic regulation, and gut–bone axis-related effects. In addition, this review discusses current challenges in processing stability, safety assessment, functional validation, and industrial application, together with future directions for the development of bone health-related functional foods and calcium nutrition strategies. It should be noted that the protein–hydrolysate–peptide continuum described in this review is intended as an analytical framework for comparing calcium-binding behavior, *in vitro* digestive behavior, and transport mechanisms across different structural scales, rather than a claim that these forms represent mutually exclusive developmental stages. In many real food applications—such as fortified yogurts, plant-based milks, or protein-enriched beverages—intact proteins, heterogeneous hydrolysates, and partially purified peptide fractions often coexist, and their interactions may produce combined calcium-binding properties that cannot be predicted from any single component alone. Recognizing this coexistence highlights the need to understand the role of each structural level before designing mixed protein–calcium chelate systems. To avoid conflating evidence levels, this review uses bioaccessibility for digestion-stage calcium release, solubility, or potential uptake; transport for epithelial transfer, uptake, permeability, or related mechanisms; absorption for *in vivo* calcium uptake or physiological absorption pathways; and bioavailability for calcium retention or utilization supported by *in vivo* evidence. Table 1 summarizes the evidence levels and interpretation of different experimental models.

**Table 1:** Evidence hierarchy of experimental models for calcium-related functional evaluation.

Model type	Mainly measures	Evidence level	Interpretation
Chemical chelation assay	Calcium-binding capacity	Screening evidence	Initial binding information
Simulated digestion	Solubility, retention, bioaccessibility	Digestive-behavior evidence	Gastrointestinal stability indicator
Cell model	Uptake, transport, transporter responses	Transport-mechanism evidence	Epithelial transport evidence
Animal model	Absorption-related indicators, bone markers	*In vivo* functional evidence	Integrated functional response
Human study	Calcium absorption, retention, bone outcomes	Clinical evidence	Human applicability

## 2 Preparation and structural basis of calcium chelates

The calcium-binding behavior and functional potential of food-derived protein-based calcium chelates depend largely on their structural hierarchy, ranging from relatively restricted binding within macromolecular protein matrices to more accessible and flexible coordination by purified peptides. Accordingly, this section discusses protein-based calcium chelates at different hierarchical levels in terms of processing and hydrolysis conditions, structural organization, accessible binding sites, coordination patterns, and stability, thereby providing a basis for subsequent discussion of *in vitro* digestive stability, intestinal transport, and bone-related effects. These preparation processes and molecular characteristics are summarized in Table 2.

**Table 2:** Preparation processes and molecular characteristics of food-derived protein-based calcium chelates at different hierarchical levels.

Source	Type	Preparation/isolation	Structural identifier	Key calcium-binding clue	References
Chicken sternum cartilage	Protein	CSCP mixed with CaCl₂ (1 mg/mL each), pH 8.0, 50 °C, 40 min	—	Ca binding sites identified at Pro14 and Glu13 in type II collagen	(19)
Egg shell/ Oyster shell/ Beef bone/ Chicken bone powder	Protein/ Protein hydrolysate	Fermentation at 30 °C for 6 days	25 kDa/75 kDa	—	(126)
Faba bean	Protein	6% dispersion, pH 6.5–7.5, overnight stirring	SDS-PAGE: legumin ~60 kDa, vicilin ~50 kDa	—	(53)
Pea bean	Protein	Same as above	SDS-PAGE: legumin ~60 kDa, vicilin ~45–50 kDa	—	(53)
Soybean	Protein	7S protein (0.4–1.6%) with CaCl₂ (0.09 M) & (NH₄)₂HPO₄ (Ca/*p* = 1.67), pH 10.0–7.6, 45 °C, 3 h,	7S subunits: *α* (~67 kDa), *α*’ (~71 kDa), *β* (~50 kDa)	—	(60)
Pea globulin	Protein	2% protein dispersion (pH 7.5), heated (70 °C–90 °C, 1 h),	SDS-PAGE: legumin (~60 kDa monomer, α ~ 38–39 kDa, β ~ 20 kDa), vicilin (~45–50 kDa)	Heat-induced soluble aggregate formation	(54)
Egg yolk	Protein hydrolysate	Defatted egg yolk protein, alkaline protease, pH 10; ultrafiltration (10, 5, 3 kDa)	EYPH1: >10 kDa; EYPH4: <3 kDa	—	(36)
Sheep bone	Protein hydrolysate	Defatted sheep bone meal (5%), alkaline protease (6,000 U/g), pH 9.5, 3 h; then neutral protease (6,000 U/g), pH 7.5, 1 h	—	—	(127)
Baby clam meat	Protein hydrolysate	Flavourzyme hydrolysis (meat/water 1:9, E/S 40 U/g, pH 7, 50 °C, 4 h), followed by ultrafiltration (30, 10, 3, 1 kDa)	<1 kDa fraction (peptide LPLTII 668.45 Da)	Small peptide-rich fraction with defined Ca-binding peptide	(67)
Soybean	Protein hydrolysate	Defatted soybean meal hydrolyzed with Alcalase (1:50, pH 9, 60 °C, 4 h), then 85% ethanol fractionation	—	—	(68)
Milk	Protein hydrolysate	Protein substrates hydrolyzed with one or two enzyme preparations (Alcalase, Promod, FlavorPro, Flavourzyme, or Formea) for 5 h under uncontrolled-pH conditions	—	Hydrolysis strategy-dependent Ca-binding behavior	(29)
Salmon frame	Protein hydrolysate	Salmon bones ground to <75 μm; hydrolyzed with Protease P1000 (~30% DH), then debittered with 2-butanol	—	—	(69)
Phosvitin	Peptide	Pretreated at 120 °C for 30 min; trypsin hydrolysis (5 U/g, pH 8.0, 8 h); DEAE anion-exchange chromatography	<1 kDa (peptides)	Representative peptides: LSKIWGR (859 Da), KFYPFG (757 Da), AEFGTEPD (864 Da)	(128)
*Chlorella pyrenoidosa*	Peptide	Alkaline protease hydrolysis (pH 9.0, 50 °C, 4 h) followed by ultrafiltration	F2: 1–3 kDa; F3: 3–5 kDa	—	(33)
Peanut	Peptide	Defatted peanut meal subjected to alkaline extraction, isoelectric precipitation, hydrolysis (alkaline protease + flavourzyme), then Sephadex G-25 and DEAE-Sepharose FF purification	Purified peptides ~992–1,692 Da	Representative peptides: NLAGNHEQEFLR, NAEFNEIR, YDEEDRRR, YEEPAQQGR, DQSQQQQDSHQKVR, NNNDNQLDQFPR	(37)
Whey	Dipeptide	Whey protein (5%) denatured at 80 °C for 20 min; hydrolyzed with Flavourzyme + Protamex (2:1, 49 °C, 7 h); purified by DEAE, Sephadex G-25, and RP-HPLC	280 Da	Phe-Asp	(23)
Phosvitin	Peptide	Partially dephosphorylated with 0.1 M NaOH (37 °C, 3 h), then trypsin hydrolysis (E/S 1:50, 37 °C, 12 h); purified by DEAE, Sephadex G-25, and RP-HPLC	1106.44 Da	DEENDQVK	(38)
Antler bone	Peptide	Flavourzyme hydrolysis (pH 8.5, 50 °C, 5 h) → chelation with antler bone Ca (malic acid extraction) at 1:1, pH 7.5, 50 °C, 60 min	567–1,649 Da	Representative collagen-derived motifs: GPGSGP, GDQGVPG, GPAGPGPP, GAPGPSGP	(44)
Casein phosphor peptides+chitosan oligosaccharides	Peptide-polysaccharide	Amadori-type CPP: COS (0.6:1, pH 8.0, 90 °C, 3 h) or TGase-type CPP: COS (1:1, TGase 15 U/g CPP, 37 °C, 2 h); then chelation with CaCl₂ (2:1, pH 7.4, 55 °C, 2 h)	COS ~ 2 kDa	Composite CPP/COS system with phosphate-containing Ca-binding domains	(42)
Lactoferrin + casein phosphopeptides	Peptide	LF: CPP mass ratio 1:3–1:4, pH 7.0, 35 °C; then mixed with CaCl₂ (1:2, w/w), 50 °C, 60 min	—	Ca binding linked to phosphate groups and Glu/Asp residues	(58)
Hericium erinaceus	Peptide	Peptide: CaCl₂ = 2:1 (w/w), pH 8.0, 50 °C, 30 min	—	Representative peptide: WYEEGTDKA; binding residues include W, E, T, D, K, A via COO^−^, N–H, and C=O groups	(129)

CSCP, chicken sternum cartilage; EYPH, egg yolk protein hydrolysate; E/S, enzyme/substrate ratio; DH, degree of hydrolysis; DEAE, diethylaminoethyl; RP-HPLC, reversed-phase high-performance liquid chromatography. Entries in this table summarize preparation processes, structural identifiers, and calcium-binding clues of food-derived protein-based calcium chelates. These data mainly correspond to preparation, screening, and structural-characterization information in the evidence hierarchy summarized in Table 1. Preparation ratios and calcium salt concentrations are listed as reported in the original studies and should not be interpreted as true calcium-binding stoichiometry or coordination number unless directly measured.

### 2.1 Protein-calcium chelates

In food-derived protein–calcium chelate systems, natural macromolecular proteins constitute the most fundamental binding matrices. Their interactions with calcium arise mainly from side-chain carboxyl groups of Asp and Glu and, in selected phosphoproteins, from phosphate groups associated with phosphoserine residues. These charge-dense sites support calcium binding through electrostatic attraction and coordination interactions (48, 49). Although the affinity of individual sites is often constrained by conformational rigidity and steric hindrance, macromolecular proteins may still display considerable apparent calcium-carrying capacity because of their large molecular size and numerous potential binding regions. However, marked source-dependent differences in coordination behavior and functional performance mean that they cannot be regarded as functionally equivalent calcium chelates.

Casein and phosvitin are representative proteins with comparatively strong apparent calcium-carrying capacity. Casein contains highly phosphorylated flexible regions that support relatively stable micellar structures with amorphous calcium phosphate, thereby maintaining calcium in a colloidally dispersed state in aqueous systems (50). Phosvitin, by contrast, contains densely clustered phosphoserine residues and can directly bind substantial amounts of calcium, resulting in pronounced local calcium enrichment (51, 52). Through these structural features, such proteins can retain relatively high levels of calcium in dispersed form while limiting macroscopic precipitation. By contrast, many food proteins, including many plant proteins and animal muscle proteins, show more limited carrier performance at the macromolecular level. For example, pea and faba bean globulins form insoluble calcium-associated aggregates at pH 6.5–7.0, with calcium binding significantly modulated by residual phytic acid (53), and heat-denatured pea globulins produce smaller, denser calcium-induced aggregates (54). In these systems, calcium binding relies mainly on relatively sparse surface-exposed carboxyl groups. As local calcium concentration increases, calcium ions may act as electrostatic bridging nodes, promoting rearrangement, hydrophobic aggregation, and intermolecular cross-linking (55, 56). At the macroscopic level, these processes may lead to phase separation, sedimentation, or dense gel formation, thereby weakening their suitability as stable calcium chelates (57).

In gastrointestinal environments, this macromolecular mode of calcium association shows a clear dual character. On the one hand, intact proteins or high-molecular-weight aggregates may temporarily retain calcium within three-dimensional networks or colloidal assemblies (58), thereby reducing direct contact with precipitation-promoting anions such as phytate, oxalate, and carbonate (48, 59). Soybean 7S/calcium phosphate chelates further show that protein–mineral assemblies can resist gastric digestion and enable intestinal release of encapsulated components, illustrating the gastric stability of such assemblies rather than directly proving improved calcium absorption (60). On the other hand, continued proteolysis by pepsin and trypsin progressively disrupts the macromolecular framework and releases bound calcium in stages. In some systems, the medium- and low molecular weight peptides generated during digestion may retain part of the original binding capacity or expose new coordination groups as steric constraints are reduced, thereby helping to maintain calcium in a soluble state (61, 62). Therefore, macromolecular proteins should not be dismissed as ineffective calcium chelates, because their performance depends strongly on protein type, native structural organization, and *in vitro* digestive behavior. However, such protective or sustained-release effects are not universal. In many macromolecular protein systems, calcium-induced aggregation and intermolecular cross-linking may hinder protease accessibility and prevent complete matrix degradation, leaving part of the bound calcium trapped in poorly digested residues (56, 63). This structural limitation reduces the applicability of proteins as general-purpose food-derived calcium chelates.

### 2.2 Protein hydrolysate-calcium chelates

Controlled enzymatic hydrolysis is an important transitional strategy for shifting calcium-binding behavior from macromolecular proteins to smaller and more accessible calcium-binding fragments. By cleaving dense macromolecular networks into mixed systems composed of polypeptides, oligopeptides, and small amounts of free amino acids, hydrolysis not only reduces steric constraints associated with protein aggregation but also exposes previously masked binding sites (13, 64). Recent studies have shown that high-throughput screening can improve substrate–enzyme matching in food-protein hydrolysis. For example, screening of whey protein isolate (WPI), milk protein isolate (MPI), and micellar casein isolate (MCI) hydrolysates prepared with commercial food-grade enzymes showed that MCI and MPI hydrolysates displayed markedly stronger calcium-, magnesium-, and iron-binding capacities than WPI hydrolysates, highlighting the importance of substrate–enzyme matching in mineral-binding performance (29). In addition, enzymes such as papain may expose not only conventional coordination sites but also nonphosphorylated active regions enriched in Glu and Lys, thereby broadening the structural basis of enzymatic modification (34, 65, 66).

In mixed hydrolysate systems, molecular weight (Mw) distribution is a key factor affecting apparent calcium-binding capacity. Release from higher-order structural constraints increases the exposure of terminal amino groups and free side-chain carboxyl groups, enabling medium- and short-chain peptides to adopt more favorable conformations for calcium coordination. However, the optimal Mw range varies among protein sources and processing conditions. In *Chlorella pyrenoidosa* protein hydrolysates, the 1–3 kDa and 3–5 kDa fractions formed relatively compact calcium-chelated ultrastructures, with digestion-stage calcium bioaccessibility reaching 58.83% and 54.74%, respectively (33). By contrast, in baby clam (*Corbiculidae* sp.) meat hydrolysates, the <1 kDa fraction exhibited the highest calcium-binding capacity, reaching 4594.87 μg/g (67). In plant protein matrices such as soy protein and wheat gluten, conventional hydrolysis alone is often insufficient because of the lack of specific natural modifications and the presence of relatively compact hydrophobic cores. Accordingly, physical pretreatment or solvent fractionation is often introduced. Ultrasound-assisted hydrolysis of wheat protein produced a more dispersed calcium-chelated structure, and quadrupole time-of-flight mass spectrometry (QTOF-MS) identified Asp as a key residue involved in calcium capture (34). Similarly, ethanol fractionation of soy protein hydrolysates yielded lower-molecular-weight alcohol-soluble fractions with stronger hydrophobicity and more pronounced metal-coordination features, as indicated by Fourier-transform infrared spectroscopy (FTIR) (68). These findings suggest that physical or chemical pretreatment combined with enzymatic hydrolysis can improve the calcium-chelating performance of some plant protein systems. Despite their functional advantages, hydrolysate–calcium systems remain compositionally heterogeneous (33, 34, 69). Sequence variation and competitive coordination can obscure the key motifs and binding patterns underlying calcium capture. This limitation has shifted increasing attention from mixed hydrolysates toward purified, sequence-defined peptides, whose amino acid sequences are identified by LC–MS/MS, peptidomics, or related approaches, thereby providing a clearer basis for analyzing calcium-coordination motifs and structure–function relationships.

### 2.3 Bioactive peptide-calcium chelates

Compared with hydrolysate systems, which are compositionally heterogeneous and difficult to characterize in terms of structure–function relationships, sequence-defined bioactive peptides provide a more suitable basis for molecular-level analysis of peptide–calcium chelation. With advances in peptidomics and high-resolution mass spectrometry, core sequences responsible for calcium coordination, together with their spatial binding modes and binding features, can now be identified more clearly (70–72). Unlike the less defined multivalent interactions in mixed systems, defined bioactive peptides generally show stronger sequence dependence and clearer structural organization in their interactions with calcium, thereby supporting sequence–structure–function analysis.

Peptide–calcium coordination is not a single binding event but can adopt different binding modes. Liquid chromatography–tandem mass spectrometry (LC–MS/MS) analysis of rabbit bone collagen-derived peptide–calcium chelates identified interlinking, loop-linking, and monolinking as the main binding patterns, with interlinking driven by acidic residues such as Asp and Glu accounting for a relatively high proportion (73). At the atomic level, density functional theory (DFT) further indicated that acidic residues with free side-chain carboxyl groups have stronger coordination tendencies and can serve as anchoring sites in highly active chelating peptides (10). FTIR and X-ray photoelectron spectroscopy (XPS) analyzes also showed that short peptides enriched in Asp/Glu mainly coordinate calcium through oxygen and nitrogen atoms in carboxyl and amino groups, forming stable multidentate structures (74, 75). Thus, the calcium affinity of bioactive peptides depends not only on the number of binding sites but also on the spatial arrangement of functional groups and their cooperative coordination modes.

Calcium coordination is often accompanied by peptide conformational reorganization. After binding calcium, peptide chains tend to adopt more compact and ordered structures and, in some systems, further assemble into nanoparticles with smaller particle sizes and narrower distributions (32, 76, 77). These findings suggest that peptide–calcium interactions involve not only local chelation but also local folding, interchain rearrangement, and higher-level structural organization (27, 78). On this basis, recent studies have used glycosylation, self-assembly, ultrasound, and high-pressure homogenization to improve structural stability and regulate nanostructure formation. Glycosylated peptides formed by the Maillard reaction with xylooligosaccharides have been reported to suppress premature calcium crystallization and improve conformational stability in complex food systems (79–81). Some bioactive peptides can also form compact ternary peptide–calcium/vitamin D₃ nanostructures, highlighting their potential as nutritional delivery platforms (82, 83).

**Figure 1:**
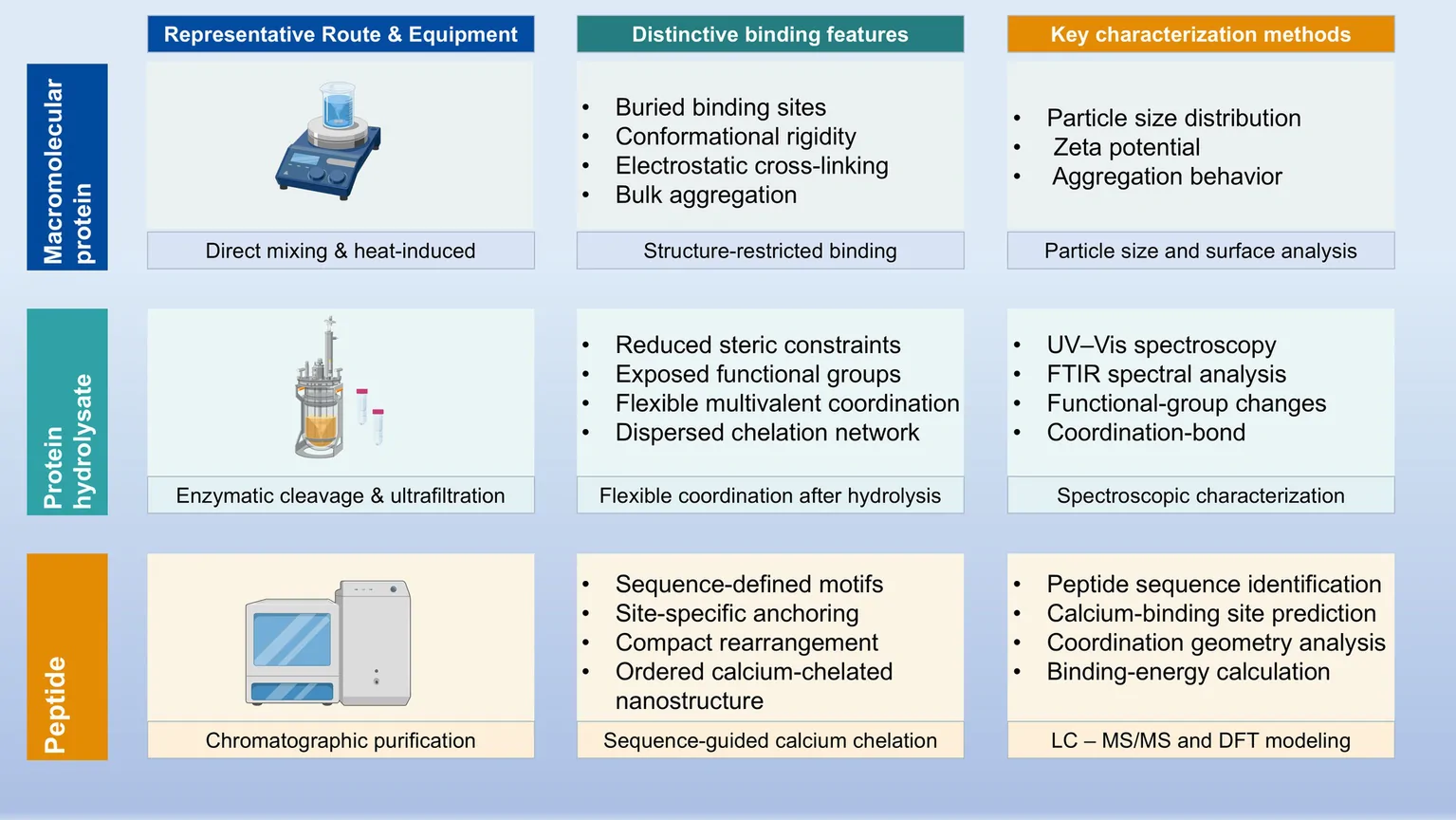
Preparation routes, binding features, and characterization methods of protein-based calcium chelates across structural hierarchies. Compiled from [Suo et al. (19); Zhang et al. (34); Liu et al. (10); Chen et al. (125)]. Created with BioGDP.com.

In addition, physical fields such as ultrasound and high-pressure homogenization further regulate microstructure by modulating local mass transfer and aggregation kinetics, thereby promoting smaller and more uniform nanostructures (34, 84, 85). However, these structural advantages do not necessarily translate into improved calcium absorption. Their stability during *in vitro* gastrointestinal digestion, interaction with the intestinal surface, and transmembrane transport mechanisms still require further clarification (see Figure 1).

## 3 *In vitro* digestive stability and gastrointestinal behavior of calcium chelates

The gastrointestinal performance of food-derived calcium chelates depends not only on their initial calcium-carrying capacity but also on their ability to maintain calcium solubility, chelated state, and bioaccessibility. Therefore, *in vitro* digestive stability and bioaccessibility are discussed as digestion-stage indicators and are not directly equated with *in vivo* bioavailability unless supported by absorption or physiological evidence. Rather than undergoing a simple binding–dissociation transition, calcium chelates experience progressive structural rearrangement in the gastrointestinal lumen, driven by proton competition and enzymatic breakdown in the gastric phase, followed by renewed coordination and competition with precipitation in the intestinal phase (86, 87). Owing to different structural hierarchies, coordination modes, and digestive stabilities, macromolecular proteins, mixed hydrolysates, and bioactive peptides exhibit distinct calcium retention, release, and solubility profiles during *in vitro* digestion. Table 3 summarizes the *in vitro* digestion-stage evidence across these structural hierarchies and should be interpreted within the evidence framework outlined in Table 1.

**Table 3:** *In vitro* digestion-stage evidence and endpoint types for gastrointestinal stability of protein-based calcium chelates across structural hierarchies.

Source	Initial Ca-binding metric	Gastric behavior	Intestinal behavior	Endpoint type	Retention mechanism	References
Chicken sternum cartilage protein	Chelation rate 76.91 ± 0.53%; optimal condition: 1 mg/mL each, pH 8.0, 50 °C, 40 min	SGF, pH 2.0; relative chelation 90.44 ± 1.04% of initial; limited proton-induced dissociation	SIF, pH 7.4; relative chelation 81.45 ± 1.97% of initial; >80% chelation maintained	Retention	Type II collagen triple helix; Pro14/Glu13 coordination; low pepsin accessibility	(19)
Egg yolk protein hydrolysate	61.31 mg/g (EYPH); 63.13 mg/g (EYPH1)	pH 2.0; EYPH–PA interaction; no Ca–PA precipitation	pH 7.0; competition with PA for Ca; reduced Ca–PA precipitation; EYPH–Ca–PA ternary chelate	Solubility	pH-dependent binary/ternary association; Ca bridge effect	(36)
Chlorella peptide	—	No significant loss of Ca-binding capacity	—	Retention	Carboxyl/amino coordination; compact structure	(33)
Peanut peptide	Chelation rate 81.4%	—	No obvious activity loss	Retention	Carboxyl/amino groups; pepsin/trypsin resistance	(37)
Whey dipeptide	73.34 mg/g	Acid phase; Ca release >95%	pH 8.0; Ca release >90%; CaCl₂ ~ 80%	Release	Carboxyl O + amide N coordination; alkaline stability; inhibition of Ca phosphate precipitation	(23)
Phosvitin peptide	151.10 mg/g	—	4 h; Ca solubility 88.91%; vs. CaCO₃ 56.43%, D-calcium gluconate 71.92%; dialysis rate 47.11%	Solubility	Carboxyl O + amino N coordination; increased β-sheet after chelation	(38)
Ethanol-soluble soy peptide	93.34 mg/g	pH 2.0; Ca-dissolution 78.65%; pH 8.0; 88.92%	*In vitro* gastrointestinal stability 71.48% after digestion	Solubility	Carboxyl O/amino N binding; Ca-induced aggregation; hydrophobic + H-bonding interactions	(68)
Baby clam peptide (LPLTII, <1 kDa)	4594.87 μg/g			Retention	Low MW; Thr4/Leu3-related binding; acid/heat stability	(67)
Amadori-type COS-CPP	—	Total Ca release ~37.6%; retained ~62.4%	4 h total release ~53.2%; retained ~46.8%; sustained-release vs. CPP–Ca	Release	COS wrapping; strong COS–Ca interaction	(42)
TGase-type COS-CPP	—	Release ~37–40%	Total release ~53%	Release	TGase cross-linked dense network; stronger CPP protection	(42)
LF-CPP-Ca	—	Ca release rate 42.38%	Ca release rate 89.43%; lower than CPP–Ca 97.91%	Release	Compact ternary structure; pepsin resistance; maintained Ca solubility	(58)
WYEEGTDKA-Ca	—	Acid-sensitive; partial Ca release	Simulated digestion; Ca retained 80.67%	Retention	Heat-stable; alkaline stability; concealed cleavage sites	(129)
Peptide-calcium chelate microcapsule	—	Slow-release rate 44.66%	Slow-release rate 51.60%	Release	β-CD wall material protection	(85)
Bovine bone peptide	Chelation rate 44.81%	Ca retention 53.32%	Ca retention 64.31%	Retention	Amino/carboxyl coordination	(130)

COS, chitosan oligosaccharide; CPP, casein phosphopeptide; LF–CPP–Ca, lactoferrin–casein phosphopeptide–calcium chelate; PA, phytic acid. Entries in this table mainly represent simulated gastrointestinal digestion evidence and correspond to digestive-behavior evidence in the hierarchy summarized in Table 1. The original endpoints were retained as reported and grouped by endpoint type to aid interpretation. These endpoints should be viewed as digestion-stage indicators, mainly including retention, release, and solubility, rather than direct measures of in vivo calcium absorption or bioavailability.

### 3.1 Stability during simulated gastric digestion

In the gastric phase, calcium chelates first encounter proton competition under low-pH conditions. In systems where carboxyl groups serve as dominant coordination sites, protonation weakens both electrostatic interactions and calcium coordination. At the same time, limited pepsin-mediated hydrolysis may alter protein or peptide backbone integrity, binding-site exposure, and spatial organization. Thus, the gastric phase should be viewed not simply as a destructive stage, but as an initial stage of structural rearrangement that influences subsequent intestinal-stage. From this perspective, the key criterion for gastric-phase performance is not whether a chelate remains completely intact, but whether it can maintain a high proportion of calcium in a bound, soluble, or dispersible form, thereby limiting premature precipitation or uncontrolled release.

For macromolecular protein–calcium chelates, gastric digestion is often accompanied by breakdown of the protein backbone. Chelates that depend on higher-order structures, micellar assembly, or gel networks for stability commonly undergo molecular-weight reduction, aggregate loosening, and exposure of previously embedded binding sites, thereby releasing part of the bound calcium. For example, dense casein micelles show characteristic coagulation behavior under acidic and enzymatic gastric conditions, and the dissociation of colloidal calcium phosphate directly affects the initial release of calcium and encapsulated components (88). However, this process depends strongly on protein source, phosphorylation level, cross-linking mode, and initial aggregation state and therefore cannot be generalized to all macromolecular systems (19). Compared with intact macromolecules, protein hydrolysates often show more stable calcium-retention behavior in the gastric phase, because their coordination depends less on complex higher-order structures and more on preparation conditions, peptide-size distribution, and exposed binding groups. For example, alkaline protease-prepared eggshell membrane hydrolysate–calcium chelates (AP-Ca) showed significantly higher calcium retention under simulated gastric conditions than systems produced by high-pressure homogenization (84). Studies on ethanol fractionation of soy, walnut, and cottonseed protein hydrolysates further showed that low molecular weight and relatively hydrophobic oligopeptide fractions were major contributors to resistance to gastric dissociation (68). Comparable trends have been reported in delivery models involving other divalent metals (Zn, Cu, and Fe), although extrapolation to calcium systems should be made with caution (88–90). Overall, the gastric stability of hydrolysates depends on peptide flexibility, multivalent coordination, and steric protection.

At the level of bioactive peptides with defined sequences, gastric stability becomes more strongly sequence dependent. DFT calculations indicate that Asp and Glu residues with free side-chain carboxyl groups can serve as thermodynamically favorable anchoring sites for calcium (10). However, such computationally inferred coordination preferences should be distinguished from experimentally validated binding mechanisms. On this basis, some screened short peptides have shown relatively strong resistance to dissociation under simulated digestion and pH conditions (25). Calcium coordination is often accompanied by clear conformational rearrangement. For example, collagen-derived peptides have been reported to shift from disordered states toward more compact and ordered structures after calcium binding, in some cases with the formation of crystalline or ultrafine nanostructures (76, 78). Such more compact coordination structures may strengthen internal binding stability and sterically hinder pepsin access. However, not all natural monomeric peptides exhibit ideal stability under strongly acidic conditions. For instance, both bovine bone collagen peptide-calcium chelates and *Moringa oleifera* leaf peptide-calcium chelates undergo pronounced dissociation during the gastric phase (91, 92). To address this limitation, glycosylated walnut peptides can enhance resistance to acid-induced dissociation by introducing steric hindrance (81). Similarly, encapsulating phosvitin phosphopeptide–calcium chelates in chitosan nanoparticles or constructing ternary assembled structures with hydrophobic peptides and vitamin D₃ can provide stronger structural protection and more controlled calcium release (82, 85, 93).

### 3.2 Stability during simulated intestinal digestion

Upon entry into the small intestine, the pH increases, allowing some coordination sites weakened in the gastric phase to re-establish calcium binding, while continued pancreatic hydrolysis further alters ligand size and conformation. The intestinal phase should therefore be regarded not simply as a continuation of gastric digestion, but as a critical stage characterized by renewed coordination, redistribution, and competition with precipitation. Because the intestinal environment contains several precipitation promoting factors, including phosphate, carbonate, and phytate, maintaining calcium in a soluble or dispersible state becomes a central determinant of intestinal phase stability. In macromolecular protein systems, relatively dense cross-linked networks can slow enzymatic degradation, thereby producing a sustained-release effect and reducing the risk of precipitation caused by transiently high local concentrations of free calcium in the lumen (61). However, this advantage appears to be system specific and should not be generalized to all macromolecular protein–calcium chelates.

In the anion-rich intestinal environment, mixed hydrolysates generally exhibit stronger competitive coordination and solubility-maintaining capacity. Peptide groups may reduce precipitation through mechanisms such as ternary chelate formation. For example, egg yolk protein hydrolysates can form soluble phytate–calcium–peptide chelates, thereby alleviating the inhibitory effect of phytate on calcium transport (36). Ultrasound-assisted wheat protein hydrolysates also maintain favorable solubility and permeability in the presence of both phytate and oxalate (34). In addition, insoluble dietary fiber may limit mineral absorption by retaining metal ions at the interface. In the zinc-based model, peptide chelation was found to weaken this retention by altering Zn adsorption at dietary fiber surfaces (94), although whether the same mechanism applies to calcium systems remains unclear. More importantly, protein hydrolysates with favorable peptide profiles not only retain a relatively high proportion of soluble calcium after *in vitro* gastrointestinal digestion but may also expose new coordination sites during further enzymatic cleavage, thereby increasing both chelating capacity and bioaccessibility (95). For example, walnut meal protein hydrolysate–calcium chelates retained high overall solubility at the end of *in vitro* intestinal digestion, while *Chlorella* hydrolysate fractions with specific molecular-weight cut-offs (<5 kDa) maintained dense calcium-chelated structures after digestion and showed markedly higher bioaccessibility than conventional inorganic calcium sources (33). In addition, the metal-chelating activity of catfish skin gelatin hydrolysates increased after gastrointestinal digestion, suggesting that further enzymatic cleavage may release previously hidden metal-affinitive sites (96). Taken together, the advantages of mixed hydrolysates lie not only in their initial binding capacity but also in their potential for structural reorganization and site re-exposure during digestion, thereby supporting calcium solubility and bioaccessibility.

Purified bioactive peptides show a different intestinal mechanism from the dynamic multicomponent behavior characteristic of mixed hydrolysates. Rather than relying primarily on dynamic multicomponent interactions, they may directly interfere with the nucleation of insoluble calcium salts through precise occupation of functional groups. Casein phosphopeptides, for example, can bind free calcium and delay the crystallization of calcium phosphate precipitates (97). Glycosylated walnut peptides have also been shown to inhibit calcium phosphate crystallization more stably (81, 98). In addition, specific peptide sequences may maintain dispersion in intestinal fluid by reshaping the interfacial properties of chelate surfaces. Silver carp skin collagen peptides can increase electrostatic repulsion at the particle surface, thereby maintaining thermodynamic stability and anti-aggregation capacity in multicomponent environments (24), whereas bamboo shoot protein peptides maintain high solubility under neutral pH and low-concentration phosphate buffer conditions through specific coordination networks involving Glu, Asp, and Lys (75). Similar phenomena have also been reported in delivery studies of other metals (7, 99, 100). For example, cattle bone collagen peptide–selenium chelates maintained high coordination stability after intestinal enzymatic digestion and showed favorable intestinal permeability (101). However, these findings mainly support a broader principle: Peptides with identified amino acid sequences, as determined by LC–MS/MS or peptidomics, may improve metal ion accessibility by inhibiting crystallization and maintaining colloidal dispersion. Their interpretation in calcium-specific systems should therefore remain cautious.

**Figure 2:**
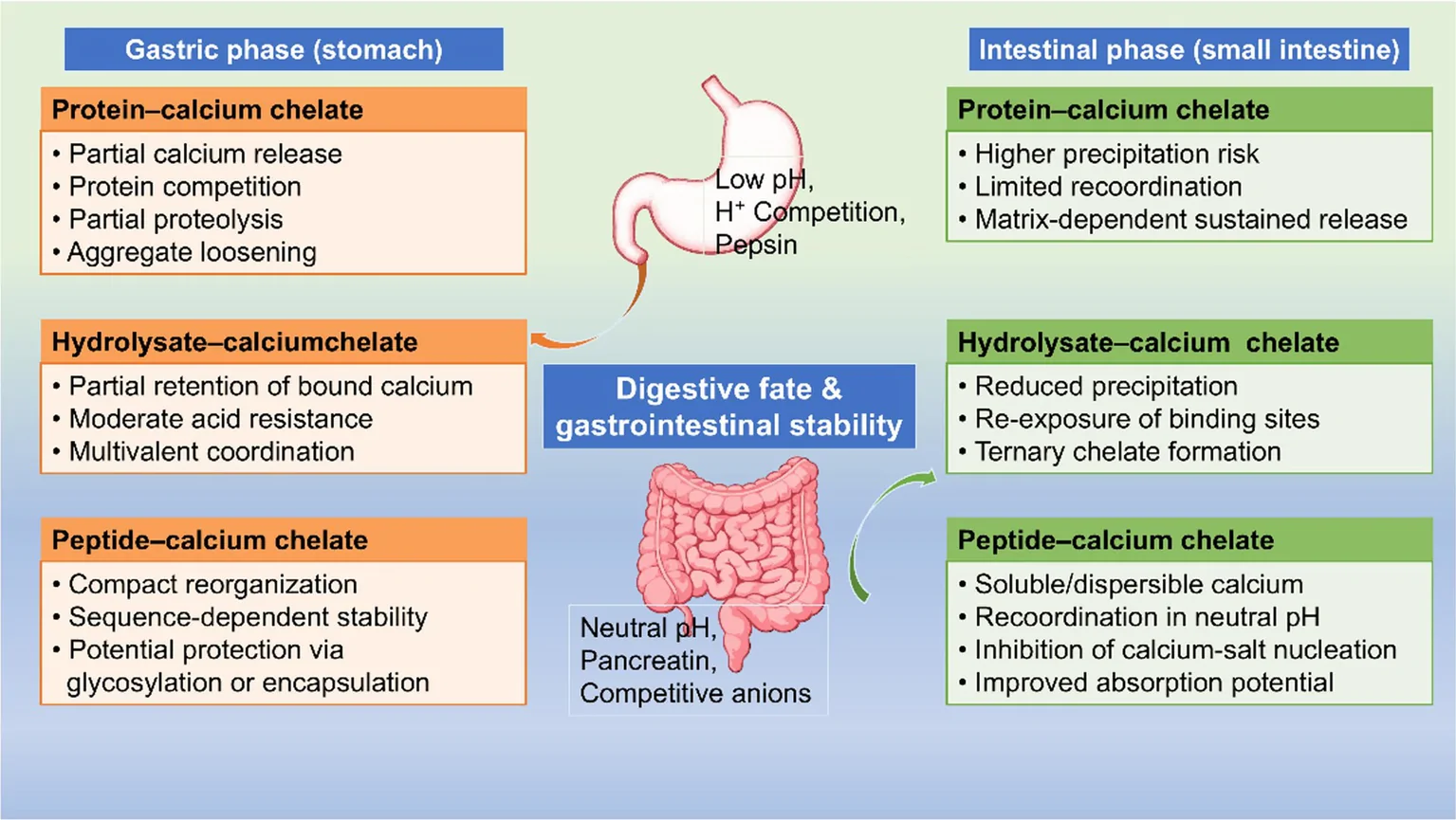
Simulated gastrointestinal stability and calcium retention behavior of protein-based calcium chelates across structural hierarchies. This figure summarizes calcium solubility, retention, and bioaccessibility under simulated gastric and intestinal conditions, rather than direct evidence of *in vivo* calcium absorption or nutritional efficacy.

Food-derived calcium chelates of different structural hierarchies differ mainly in their capacity to retain calcium and resist precipitation during *in vitro* gastrointestinal digestion. Macromolecular protein systems provide abundant initial calcium-binding sites and a certain degree of structural protection, but they are also more susceptible to structural depolymerization during digestion and may therefore face a higher subsequent risk of precipitation, although this behavior remains strongly system dependent. Mixed hydrolysates combine structural flexibility with multivalent coordination advantages and, in some systems, may further enhance calcium retention through re-exposure of coordination sites; however, their compositional heterogeneity makes structure–function relationships difficult to define precisely. By contrast, bioactive peptides with defined sequences are more suitable for mechanistic analysis of the links between *in vitro* digestive behavior and calcium retention, especially in the intestinal phase, where they may maintain calcium in soluble or dispersible states through competitive coordination, inhibition of precipitation nucleation, or improved interfacial dispersion stability (48, 58). As summarized in Table 3, food-derived calcium chelates across structural levels exhibit distinct calcium-retention and solubility-maintaining behaviors under simulated gastric and intestinal conditions. These characteristics help explain their gastrointestinal stability, but should be interpreted as prerequisites for subsequent calcium absorption and utilization, rather than as direct evidence of nutritional efficacy. Moreover, most available evidence is derived from simulated digestion systems, which are useful for controlled comparison but cannot fully reproduce the dynamic complexity of human digestion. Whether these chelates can be efficiently absorbed across the intestinal epithelium and ultimately contribute to bone-related functions still depends on a deeper understanding of their transport pathways and *in vivo* behavior (see Figure 2).

## 4 Intestinal transport and gut–bone axis regulation

The bone-related effects of food-derived protein-based calcium chelates depend not only on calcium solubility in the intestinal lumen, but also on their capacity to support epithelial calcium uptake or transport and influence systemic bone metabolism after absorption. Their physiological role has therefore expanded from simple mineral carriage to a broader framework involving local solubilization, transmembrane transport, and postabsorptive regulation of bone-related pathways. Table 4 summarizes the available evidence on intestinal transport, bone-related outcomes, and gut–bone axis-related responses.

**Table 4:** Transport evidence, bone-related outcomes, and gut–bone axis responses of protein-based calcium chelates across study models.

Type	Source	Evidence level	Transport / bone-related outcome	Gut–bone axis	References
Protein	Chicken sternum cartilage	*In vitro* (MC3T3-E1 subclone 14 cell)	Promoted osteoblast differentiation and mineralization; increased ALP, OCN, Col-I, and mineralized nodules	—	(19)
Protein	Chicken sternum cartilage	*In vivo* (low-calcium-fed SD rats)	Increased BMD; improved trabecular microstructure; increased serum OCN and Col-I; decreased ALP and bone loss	Increased α-diversity, Bifidobacterium, Roseburia, and Faecalibacterium; decreased Helicobacter, Desulfovibrio, and Escherichia-Shigella; increased SCFAs	(19)
Peptide	Egg yolk phosvitin	*In vitro* (Caco-2 cell)	Upregulated Calbindin-D9k mRNA expression (2.6-fold of control)	—	(128)
Protein hydrolysate	Sheep bone	*In vivo* [SD rat (low-calcium diet, 8 weeks)]	Increased bone calcium content (191.88 vs. 176.64 mg/g), BMD (0.19 vs. 0.06 g/cm^3^), BV/TV, and Tb.Th; decreased Tb.Sp; improved bone mechanical strength; reduced ALP, OCN, DPD, TRACP-5b, and β-CTX	Increased *Firmicutes; decreased Proteobacteria* and *Verrucomicrobiota*; increased acetate, propionate, and butyrate	(127)
Peptide	*Chlorella pyrenoidosa*	*In vitro* (Caco-2 /RAW 264.7 cell)	Protected RAW264.7 cells from H_2_O_2_-induced oxidative stress; increased CAT and SOD activities	—	(33)
Peptide	Peanut peptide (PPH21-Ca)	*In vitro* (Caco-2 cell)	Bioaccessibility 58.4% vs. 37.0% for CaCl_2_; concentration- and time-dependent	—	(37)
Peptide	Casein phosphopeptides	*In vitro* (Caco-2 cell)	Increased total calcium transport at 30 min (2 mg/mL, *p* < 0.01); increased TRPV6, occludin, and ZO-1 expression; two CPPs detected in basolateral chamber	—	(62)
Peptide	Antler bone	*In vitro* (Caco-2 cell)	Increased calcium transport in Caco-2 cells (3.9-fold at 30 min)	—	(44)
Peptide	Antler bone	*In vivo* (ICR mouse (D-gal induced aging))	Increased serum calcium (63.6 vs. 38.1 μmol/dL) and P1NP; decreased ALP and CTX-I; increased tibia calcium and BMD; improved bone microstructure	—	(44)
Peptide–oligosaccharide conjugate	Amadori-type COS-CPP/ TGase-type COS-CPP	*In vitro* (Caco-2 cell)	Increased intracellular calcium (change 154.4/166.3 vs. 52.6 for CaCl_2_)	—	(42)
Peptide–oligosaccharide conjugate	TGase-type COS-CPP/ Amadori-type COS-CPP	*In vivo* [Kunming mouse (low Ca diet)]	Increased serum calcium; decreased ALP; increased bone strength and bone weight; improved BV/TV and Tb.Th; decreased Tb.Sp and Tb.Pf	Increased *Bifidobacterium* and *Lactobacillus*; decreased *E. coli* and *C. perfringens*; increased caecal butyrate	(42)
Ternary peptide	Lactoferrin + CPP	*In vitro* (Caco-2 cell)	Calcium transport ~2.8 μg/well at 120 min; reduced inhibition by phytic acid and oxalic acid.	—	(58)

ALP, alkaline phosphatase; OCN, osteocalcin; Col-I, type I collagen; BMD, bone mineral density; BV/TV, bone volume/tissue volume; Tb.Th, trabecular thickness; Tb.Sp, trabecular separation; Tb.Pf, trabecular pattern factor; DPD, deoxypyridinoline; TRACP-5b, tartrate-resistant acid phosphatase 5b; β-CTX, beta C-terminal telopeptide of type I collagen; CTX-I, C-terminal telopeptide of type I collagen; P1NP, procollagen type I N-terminal propeptide; CAT, catalase; SOD, superoxide dismutase; SCFAs, short-chain fatty acids. Evidence levels indicate whether the reported outcomes were obtained from *in vitro* cell models or *in vivo* animal models, according to the hierarchy summarized in Table 1.

### 4.1 Transport pathways

Intestinal absorption is a critical step in determining calcium utilization. The brush border of intestinal epithelial cells and the tight-junction barrier together form the principal physical and biological interface for calcium uptake. Because calcium chelates differ in molecular size, coordination mode, and interfacial behavior, they do not follow a single absorptive route. Although macromolecular proteins can initially protect calcium, extensive gastrointestinal proteolysis ultimately releases sequestered calcium for conventional free-ion absorption. In the duodenum, this process depends mainly on the active and saturable uptake of calcium through transient receptor potential vanilloid 6 (TRPV6), which is tightly regulated by vitamin D (102, 103), whereas passive paracellular diffusion in the distal intestine is constrained by tight-junction pore size (104) and luminal transit time (105). However, the free-ion model faces clear physiological limitations. In the alkaline environment of the small intestine, free calcium readily forms poorly absorbable precipitates with dietary antagonists such as phytate (36). Together with the saturable nature of TRPV6-mediated uptake, these constraints suggest that classical free-ion transport cannot fully explain the improved calcium uptake or absorption-related responses observed in some protein–calcium chelate systems and therefore point to additional pathways involving local protection of calcium and non-classical epithelial transport.

Compared with intact proteins, mixed hydrolysates generated by enzymatic digestion often show more pronounced interfacial advantages in promoting calcium absorption. Short peptide fragments expose more carboxyl, amino, and phosphate groups, thereby maintaining calcium in a more soluble or finely dispersible state and increasing the effective calcium substrate at the brush border surface (15, 76). In the Caco-2 cell model, AP-Ca showed markedly higher transport and permeability than inorganic calcium and intact protein–calcium chelates (84). However, the superior permeability of hydrolysate–calcium chelates cannot be attributed to improved interfacial behavior alone. Specific bioactive peptides within hydrolysate mixtures have also been reported to upregulate epithelial calcium transport proteins, particularly transient receptor potential vanilloid 5 (TRPV5) and TRPV6 (39, 66), while some digestion-released sequences appear able to interact with the Venus flytrap domain of the calcium-sensing receptor (CaSR), thereby stimulating calcium transport machinery (92). Thus, the transport advantage of protein hydrolysates appears to arise from the combined effects of improved luminal solubility, increased paracellular permeability, and activation of transcellular transport pathways.

High-affinity short peptides and modified systems may display a more sequence-directed mode of calcium transport that goes beyond classical free-ion absorption. Molecular docking and molecular dynamics analyzes have shown that the soybean peptide GGDLVS not only upregulates key transport proteins, including TRPV6, calbindin-D9k, plasma membrane calcium ATPase 1b (PMCA1b), and sodium/calcium exchanger 1 (NCX1), but also forms hydrogen-bonding networks with TRPV6, thereby stabilizing its open conformation and promoting calcium enrichment at the channel entrance (106). Glycosylated walnut peptide COS-AERGVLYR-Ca has also been reported to enhance transcellular transport through activation of the voltage-dependent calcium channel CaV1.3 (98), whereas transport of the silver carp skin collagen peptides–calcium chelate (SSCPs-Ca) appears to occur independently of vitamin D (24). In addition, some peptide systems seem to operate through dual-pathway synergy. For example, whey protein peptide–calcium chelates were reported to increase paracellular permeability while simultaneously upregulating TRPV6, Calbindin-D9k, and PMCA1b to promote transcellular transport (58). In calcium-deficient mice, an oyster-derived peptide–calcium chelate significantly restored impaired ileal architecture and increased villus surface area and goblet cell proliferation compared with calcium carbonate (65). These findings support a broader view in which calcium-binding chelates improve intestinal uptake not only by maintaining luminal calcium in an available state, but also by modulating epithelial transport machinery and mucosal structure.

Overall, intestinal uptake of calcium-binding chelates should not be understood solely as free calcium supply. Current evidence instead supports a multilevel framework involving local solubilization, barrier modulation, and the combined action of multiple transport pathways. Improved transmembrane transport, however, should be viewed as a prerequisite for subsequent physiological effects rather than the final outcome. At present, mechanistic studies still rely heavily on Caco-2 monolayers and therefore only partially reflect the intestinal environment *in vivo*. Intestinal enteroids and other microphysiological intestinal models may help clarify the transport and barrier behavior of food-derived calcium chelates.

### 4.2 Osteogenic signaling and gut microbiota remodeling

After intestinal absorption, the potential bone-related effects of peptide–calcium chelates are not limited to calcium supply for bone mineralization. Available evidence indicates that these chelates may further influence signaling pathways associated with osteoblast activity, osteoclast activity, and inflammatory regulation (80, 81). Their potential bone-protective effects may involve two interconnected dimensions: direct regulation of osteogenic networks and indirect modulation of the gut–bone axis. At the osteogenic signaling level, some peptide–calcium chelates have been associated with activation of CaSR-related responses, thereby engaging the classical Wnt/*β*-catenin pathway (92) and modulating both FGF2/RUNX2-associated osteogenesis and the OPG/RANKL axis involved in osteoclast inhibition (87). In line with these observations, *in vivo* models of osteoporosis and calcium deficiency demonstrate that these chelates upregulate osteogenic markers, suppress resorption, and improve trabecular microarchitecture and biomechanical strength (87, 107).

Indirectly, peptide–calcium chelates may modulate gut microbiota composition and reduce low-grade inflammation. Interventions with specific chelates, (e.g., from walnut and tilapia) enrich beneficial taxa such as *Bifidobacterium*, *Bacteroides*, *Akkermansia*, and *Lactobacillus* (35, 87, 91). These microbial shifts are accompanied by increased bone mass and improved bone metabolic markers. Current evidence further suggests that such gut microbiota changes may exert bone-protective effects by promoting SCFAs production, reducing gut-derived inflammatory burden, and modulating OPG/RANKL/RANK-related signaling. In intestinal epithelial–osteoblast–osteoclast three-cell coculture models, polypeptide intervention has been shown to alter intercellular communication and downregulate signaling molecules related to bone resorption (108). *In vivo*, similar effects are accompanied by reduced circulating levels of proinflammatory factors such as TNF-α and IL-6 (109). These findings indicate that peptide–calcium chelates may contribute to bone health not only by improving mineral availability, but also by affecting intestinal and bone-related signaling pathways.

However, a fundamental challenge in interpreting the gut microbiota-related findings associated with peptide–calcium chelates is the difficulty of isolating the contributions of the peptide component, free calcium ions, and the intact chelate form. Calcium itself is known to modulate microbial ecology: luminal calcium can precipitate bile acids and fatty acids, reducing their cytotoxic effects on colonocytes and selectively favoring the growth of certain commensal bacteria (7, 110). Similarly, SCFAs production following peptide–calcium chelate administration may partly reflect fermentation of residual peptide fragments by colonic microbiota, rather than a specific effect of the chelate structure (111). The observed enrichment of *Bifidobacterium* and *Lactobacillus* populations, while consistently reported, has rarely been tested against a “free peptide + free calcium” control group that would allow these contributions to be separated. Without such comparisons, it remains unclear whether the chelate format confers any microbiota-modulatory advantage beyond what equivalent doses of calcium salt or hydrolysate alone would produce.

The attenuation of pro-inflammatory markers, including TNF-*α* and IL-6, similarly lacks mechanistic specificity in the current literature. Calcium supplementation alone has been reported to suppress intestinal inflammation through modulation of tight junction proteins and NF-κB signaling (112), while bioactive peptides derived from food proteins may independently exert anti-inflammatory effects through ACE inhibition or opioid receptor interactions (113, 114). The degree to which peptide–calcium chelates produce synergistic, additive, or merely overlapping anti-inflammatory effects relative to their individual components has not been systematically evaluated.

**Figure 3:**
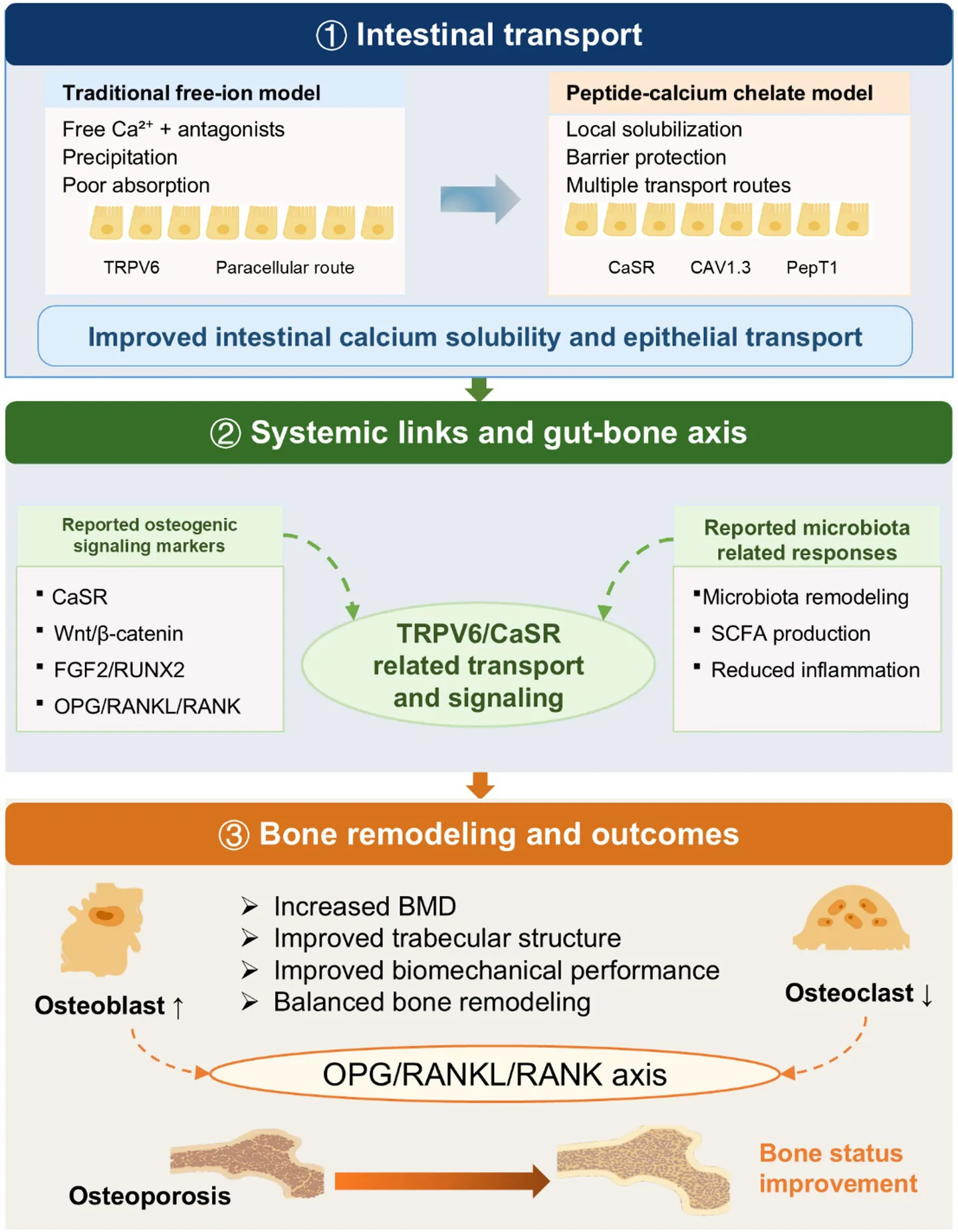
Intestinal transport and gut–bone axis regulation of protein-based calcium chelates. All pathways, markers, and outcomes shown have been reported in experimental studies. Transport-related markers, osteogenic signaling markers, microbiota-related responses, and bone remodeling-related outcomes are presented as different evidence categories and should not be interpreted as a fully validated causal sequence. Created with BioGDP.com.

From a methodological standpoint, most available evidence derives from rodent models using highly purified chelate preparations under controlled dietary conditions, which limits translation to complex food matrices where chelate integrity, competing matrix components, and microbial community composition may differ substantially. Clinical intervention studies directly examining gut microbiome responses to peptide–calcium chelates remain insufficient. Future studies could incorporate multi-arm designs including calcium salt control, free peptide control, physical mixture of free peptide and calcium salt, and intact peptide–calcium chelate treatment at equivalent calcium and nitrogen doses. Combined with metagenomic sequencing and SCFA profiling, such comparisons would help determine whether the intact chelate produces effects distinct from, or greater than, those of its individual components, thereby clarifying the specific contribution of the chelate structure to gut–bone axis-related responses (see Figure 3).

### 4.3 Vitamin D dependency and partial vitamin D-independent absorption of peptide–calcium chelates

Calcium absorption in the small intestine proceeds through two mechanistically distinct routes whose different dependence on vitamin D status has direct bearing on the interpretation of peptide–calcium chelate function (115). The transcellular active transport pathway, which predominates under conditions of low luminal calcium concentration or high physiological demand, is tightly regulated by 1,25-dihydroxyvitamin D₃ (calcitriol) through transcriptional upregulation of the apical calcium channel TRPV6, the intracellular carrier calbindin-D9k, and the basolateral extrusion proteins PMCA1b and NCX1 (103). In contrast, passive diffusion through tight junction complexes, partially mediated by claudin-2 and claudin-12, is mainly driven by luminal calcium concentration and electrochemical gradient, and is less directly dependent on vitamin D signaling, although vitamin D can regulate the expression of tight junction proteins at physiological concentrations (116, 117).

Peptide–calcium chelates interact with both pathways in ways that confer partial, but not complete, independence from vitamin D status. One proposed mechanistic feature of peptide-bound calcium transport is the possible involvement of peptide transporter 1 (PepT1) and related oligopeptide transporters on the apical membrane, which operate through a proton-coupled mechanism and are regulated mainly by luminal pH and substrate affinity rather than vitamin D (118). If intact or partially intact peptide-bound calcium species are transported through this route in selected systems, they may reduce reliance on the calcitriol-regulated transcellular calcium transport machinery; however, the quantitative contribution of this pathway remains uncertain (119, 120). Additionally, by maintaining calcium in a soluble or chelated form throughout the gastrointestinal tract—reducing precipitation as insoluble calcium phosphate or calcium oxalate in the alkaline intestinal environment: peptide–calcium chelates may increase the fraction of calcium available for passive paracellular absorption in the distal small intestine and proximal colon, where vitamin D-dependent active transport is less prominent (87, 103, 104). These combined properties suggest that peptide–calcium chelates may provide an absorptive advantage over inorganic calcium salts under conditions of suboptimal vitamin D status, but this advantage should not be interpreted as complete vitamin D independence (121).

However, several important caveats temper this conclusion. First, PepT1-mediated transport of intact peptide–calcium chelates represents only a fraction of total calcium uptake, and its quantitative contribution relative to transcellular and paracellular routes has not been directly measured in humans under controlled vitamin D conditions (103, 118). Second, severe vitamin D deficiency is associated with diffuse intestinal mucosal changes including reduced epithelial surface area, altered tight junction composition, and impaired enterocyte differentiation that would be expected to compromise all absorption routes, including those nominally independent of calcitriol signaling (104, 122). Third, even if intestinal absorption is partially preserved in vitamin D-deficient states, the downstream utilization of absorbed calcium for bone mineralization depends on coordinated osteoblast activity and renal calcium handling, both of which are substantially regulated by vitamin D (82, 122). A chelate that delivers calcium to the portal circulation more efficiently is of limited skeletal benefit if the hormonal environment for bone formation remains impaired (103). Critically, the question of whether peptide–calcium chelates can meaningfully decouple calcium absorption from vitamin D status has not been tested directly in well-controlled clinical trials stratified by vitamin D sufficiency. Existing animal evidence suggests that bioactive peptide calcium chelates can partially compensate for reduced active transport capacity, as demonstrated by vitamin D receptor knockout or dietary vitamin D restriction models (123). However, the extent and clinical relevance of this compensation in populations with widespread vitamin D deficiency (serum 25(OH)D between 12 and 20 ng/mL) remain unknown (124). This represents a strategically important knowledge gap, given that vitamin D deficiency and calcium insufficiency frequently co-occur in the populations most likely to benefit from enhanced calcium delivery systems, including postmenopausal women, older adults, and individuals with limited sunlight exposure or malabsorptive conditions.

On balance, current mechanistic evidence supports the view that peptide–calcium chelates can enhance calcium absorption through pathways that are at least partially independent of vitamin D, and may therefore offer a relative advantage over conventional calcium salts in vitamin D-insufficient individuals. Nevertheless, adequate vitamin D status should be considered a prerequisite for maximizing chelate efficacy, and future clinical trials of peptide–calcium chelates should stratify participants by baseline 25(OH)D levels and report outcomes separately by vitamin D status. Evaluation of peptide–calcium chelates with or without vitamin D supplementation in deficient populations may provide a more realistic strategy for clarifying their contribution to calcium delivery and bone-related nutritional outcomes.

## 5 Challenges and future prospects

### 5.1 Strategies for the industrial and clinical translation of peptide–calcium chelates

Although protein-based calcium chelates show considerable potential, their translation into commercial products still faces substantial processing, safety, and regulatory challenges. Bridging the gap between basic research and industrial application will require an integrated development strategy spanning material design, processing compatibility, product standardization, safety assessment, functional validation, and regulatory assessment. During large-scale processing in multicomponent food matrices, macromolecular proteins and mixed hydrolysates may undergo thermal aggregation, moisture-induced instability, and the development of bitter off-flavors. High-purity peptide–calcium chelates are further challenged by heat exposure, freeze-induced stress during dehydration, and competitive interactions with matrix components such as polyphenols and tannins, which may disturb coordination structures or peptide integrity. Future formulation design therefore needs to move beyond conventional sodium alginate or liposomal delivery systems toward more robust food-compatible delivery systems. For example, incorporation of peptide–calcium chelates into plant-derived nanocellulose-stabilized Pickering emulsions or composite hydrogels may improve resistance to processing stress and gastric degradation while also masking undesirable sensory attributes. In addition, carrier selection should be matched to product type and application scenario: relatively low-cost macromolecular systems may be suitable for bulk fortification, more soluble mixed hydrolysates may be better suited to clean-label beverages, and sequence-defined high-purity chelates may be more appropriate for high-value formulations, including foods for special medical purposes. From a practical formulation perspective, carrier–product matching should go beyond nominal calcium-binding capacity and consider effective calcium delivery. For a representative partial supplementation target of approximately 300 mg Ca per serving, most higher-loading chelates summarized in Table 3 would theoretically require only gram-level material, but this estimate should be interpreted together with gastrointestinal retention, solubility, bioaccessibility, epithelial transport, sensory acceptability, cost, and validated bone-related outcomes.

Beyond formulation feasibility and processing compatibility, safety assessment should also be integrated into the translational development of protein-based calcium chelates. Although many proteins, hydrolysates, and peptides have been evaluated for antioxidant, anti-inflammatory, osteogenic, or mineral-transport activities, such evidence cannot fully replace targeted safety evaluation when these materials are used as calcium-chelating carriers. Key concerns include raw-material quality, actual calcium content, dosage safety, allergenicity, and individual variability. In particular, heavy metal contamination and other residues should be carefully controlled for carriers derived from bones, shells, marine by-products, algae, and plant proteins. Industrial products should also report calcium loading capacity, free calcium fraction, and calcium-retention stability, rather than relying only on chelation rate or structural characterization. Because these carriers differ widely in source, peptide composition, calcium content, and intended intake, maximum tolerated intake or no-observed-adverse-effect level should be evaluated on a formulation-specific basis. Allergenicity should also be considered for carriers derived from milk, egg, fish, shellfish, soy, wheat, peanut, and other allergenic proteins, since enzymatic hydrolysis may not completely eliminate immunoreactive sequences.

At the same time, the field needs to move beyond empirical hydrolysis optimization toward more rational design frameworks. Integration of quantitative structure–activity relationship models with molecular docking and molecular dynamics simulations may support high-throughput virtual screening of candidate peptides and facilitate prediction of coordination geometry and binding free energy. However, computational design alone will not be sufficient. A continuous validation pathway linking molecular characterization, *in vitro* digestive stability, transport behavior, safety evaluation, and clinical validation remains essential. Future clinical randomized controlled trials should incorporate sensitive bone turnover markers, such as CTX-1 and P1NP, rather than relying solely on transient changes in serum calcium. In parallel, because interindividual variation in the gut microbiota may influence gut–bone axis responses, co-delivery strategies combining prebiotics, probiotics, and peptide–calcium systems may represent a potential direction for individualized nutritional strategies in bone health.

A question that naturally arises from the continuum framework is whether future calcium nutrition strategies should converge on single, sequence-defined peptide–calcium chelates or embrace deliberately engineered mixtures that integrate multiple structural tiers. Single well-defined chelates offer substantial advantages for mechanistic research and clinical nutrition applications. Their defined coordination geometry, predictable digestive behavior, and traceable transport pathways make them suitable candidates for establishing dose–response relationships and obtaining regulatory approval as functional ingredients or medical nutrition products. In contexts where calcium deficiency is severe—such as osteoporosis management or post-surgical nutritional support—the ability to deliver a structurally characterized, bioactive calcium chelate with documented efficacy may justify the higher cost of purification and quality control.

However, for the broader domain of functional food development, engineered mixtures may represent a more pragmatic and potentially useful strategy. Heterogeneous systems derived from controlled enzymatic hydrolysis naturally contain a spectrum of peptide lengths and coordination motifs, some of which may act synergistically: longer peptides may support initial solubilization and protect shorter bioactive sequences from aggregation, while low molecular weight chelates drive epithelial transport. Furthermore, the presence of intact protein fractions may provide a reservoir that releases calcium-binding peptides progressively during gastrointestinal digestion, effectively extending the absorptive stage. This temporal release characteristic of mixed systems may be difficult to replicate with a single purified peptide and may actually represent a functional advantage in real food matrices.

In practical implementation, two parallel paths can be adopted: on the one hand, well-defined chelates can be selected as a reference standard and detection tool to help identify which structural features are most important for biological activity. On the other hand, data-driven mixture design based on predictive models and high-throughput screening can translate these findings into formulations that are both cost-effective and practically applicable. Advances in peptidomics, machine learning-assisted peptide selection, and process engineering are beginning to make this bidirectional translation feasible. Ultimately, the choice between purity and complexity will depend on the intended application, target population, food matrix constraints, safety requirements, and regulatory environment, and both strategies are likely to coexist as complementary directions in future calcium nutrition research.

### 5.2 The underdeveloped ADME profile of peptide–calcium chelates

A recurring limitation in the peptide–calcium chelate literature is the near-exclusive focus on gastrointestinal behavior at the expense of post-absorptive absorption, distribution, metabolism, and excretion (ADME) characteristics. While several animal studies have reported elevated serum calcium concentrations, improved femoral BMD, and increased tibial calcium content following oral administration of peptide–calcium chelates relative to inorganic calcium salts or calcium-free controls, these measurements are typically reported as static endpoints rather than as components of a dynamic pharmacokinetic profile. Time-course data capturing peak serum calcium, area under the curve (AUC), and tissue distribution coefficients are rarely reported, making it difficult to assess whether the apparent advantages of chelate formats reflect enhanced absorption rate, improved retention, or reduced urinary excretion.

The metabolic fate of the peptide ligand itself represents a particularly neglected dimension. Upon intestinal absorption, the peptide moiety enters systemic circulation and is subject to rapid proteolytic degradation, renal filtration, and hepatic metabolism. Whether any intact peptide–calcium chelate survives to reach target tissues such as bone, kidney, or liver, and whether the peptide exerts its reported osteogenic signaling effects systemically or exclusively at the intestinal epithelium, remains unresolved. This distinction has important implications for understanding mechanism: if bioactivity is confined to the gut lumen and epithelial surface, then osteogenic effects must be mediated indirectly through gut–bone axis signaling rather than through direct action on osteoblasts.

Urinary calcium excretion, while sometimes used as a surrogate for net absorption efficiency, is itself a composite outcome influenced by renal tubular reabsorption, parathyroid hormone status, and vitamin D signaling, all of which may be independently affected by dietary protein load. Few studies have used stable isotope tracing (e.g., ^44^Ca or ^43^Ca) to directly quantify fractional calcium absorption from peptide–calcium chelates *in vivo*, which remains a robust approach for discriminating true absorptive enhancement from post-absorptive redistribution.

Closing this ADME gap will require multi-compartment study designs incorporating isotope-labeled chelates, serial blood sampling, tissue digestion assays, and urinary mineral profiling, ideally in both animal models and human volunteers. Integration of metabolomics approaches to track peptide ligand degradation products in plasma and urine would further clarify the systemic fate of the carrier component. Until such data are available, claims regarding improved calcium absorption-related performance of peptide–calcium chelates should be interpreted cautiously and limited to the specific endpoints measured, while their broader ADME profile remains insufficiently characterized.

### 5.3 Future prospects

The current evidence base for peptide–calcium chelates rests heavily on *in vitro* digestion models and rodent feeding studies, with human randomized controlled trial (RCT) data remaining virtually absent from the literature. While animal models have provided valuable mechanistic insights into chelate stability, transporter-mediated uptake, and osteogenic signaling, the translation of these findings to human physiology cannot be assumed. Rodent intestinal anatomy, transit time, microbiome composition, and calcium homeostatic regulation differ substantially from those in humans, and effect sizes observed in animal studies frequently do not predict human responses with acceptable precision.

If the field is to prioritize a single human-relevant endpoint for investigation over the next 3 to 5 years, fractional calcium absorption (FCA) measured by stable isotope dual-label methodology should be considered a particularly important target. FCA directly quantifies the proportion of ingested calcium that crosses the intestinal epithelium and enters systemic circulation, making it the most mechanistically proximate human endpoint to the absorptive processes discussed throughout this review. Using paired stable isotopes—typically an oral dose of ^44^Ca or ^43^Ca administered with the chelate and an intravenous reference dose of ^42^Ca—FCA can be determined from urine or serum enrichment ratios within a study stage of 24–72 h, making it logistically feasible within the timeframe of a standard clinical intervention study. Critically, this methodology allows direct comparison of chelate formats against equimolar calcium salt controls and free peptide controls within the same subject, providing the within-person attribution resolution that cross-sectional or between-group designs cannot offer.

The priority of FCA over longer-term endpoints such as BMD or bone turnover markers stems from several considerations. First, FCA is the rate-limiting determinant of downstream calcium utilization; demonstrating a reproducible FCA advantage in humans would provide the mechanistic justification needed to power adequately sized BMD trials, which typically require 2 to 3 years of follow-up and hundreds of participants to detect clinically meaningful differences. Without human FCA data, the field risks investing substantial resources in underpowered BMD trials whose null results would be uninterpretable—reflecting either true absence of effect or insufficient absorptive advantage to manifest as measurable bone accrual. Second, FCA is sensitive enough to detect meaningful differences over intervention periods of 4 to 8 weeks, making it tractable for proof-of-concept studies in high-priority populations such as postmenopausal women, older adults with habitual low calcium intake, and individuals with calcium malabsorption syndromes. Third, establishing human FCA benchmarks for specific peptide–calcium chelate structures would directly validate the *in vitro* and rodent transport data reviewed here, creating a translational bridge that currently remains insufficiently developed.

Beyond FCA, secondary endpoints in such trials should include serum parathyroid hormone (PTH) and 1,25-dihydroxyvitamin D as indicators of calcium homeostatic response, urinary calcium-to-creatinine ratio as a practical absorptive surrogate, and where feasible, gut microbiome profiling to assess whether observed microbiota shifts in animal models replicate in humans. However, FCA should remain the primary, pre-specified endpoint around which trials are powered, ensuring that the fundamental absorptive claim of the peptide–calcium chelate concept receives the rigorous human-level test it currently lacks.

## 6 Conclusion

This review outlines a cross-scale analytical framework linking molecular conformation, *in vitro* digestive stability, intestinal transport, and bone-related signaling to describe the structural and functional progression of protein-based calcium chelates, from macromolecular protein–calcium chelates to protein hydrolysate–calcium chelates and sequence-defined peptide–calcium chelates. This progression involves not only molecular-size reduction, but also changes in coordination behavior, calcium retention, digestive stability, and epithelial transport. Macromolecular protein–calcium chelates act as foundational carriers, but their performance may be constrained by steric hindrance, digestive depolymerization, and system-dependent calcium release or precipitation. Protein hydrolysate–calcium chelates represent an important transitional form, supporting calcium solubility and epithelial transport in some systems through exposed coordination sites and greater structural flexibility. Sequence-defined peptide–calcium chelates provide a more suitable basis for mechanistic analysis because they allow clearer interpretation of coordination geometry, transport behavior, and bone-related effects. In selected systems, these peptides may contribute to calcium delivery, osteogenic signaling, and gut–bone axis-related pathways, although such effects still require further validation in physiologically relevant models and well-designed clinical studies. In addition, safety assessment, including raw-material quality control, actual calcium content, dosage safety, allergenicity, and individual variability, should be integrated into future translational development. Overall, this review supports a structurally informed framework for evaluating food-derived protein-based calcium chelates, moving calcium nutrition research beyond simple mineral supplementation toward more mechanism-guided and application-specific strategies.
